# Emerging Therapeutic Targets Against *Toxoplasma gondii*: Update on DNA Repair Response Inhibitors and Genotoxic Drugs

**DOI:** 10.3389/fcimb.2020.00289

**Published:** 2020-06-12

**Authors:** Sergio O. Angel, Laura Vanagas, Diego M. Ruiz, Constanza Cristaldi, Ana M. Saldarriaga Cartagena, William J. Sullivan

**Affiliations:** ^1^Laboratorio de Parasitología Molecular, Instituto Tecnológico Chascomús (INTECH), Consejo Nacional de Investigaciones Científicas (CONICET)-Universidad Nacional General San Martin (UNSAM), Chascomús, Argentina; ^2^Pharmacology and Toxicology, Indiana University School of Medicine, Indianapolis, IN, United States; ^3^Microbiology and Immunology, Indiana University School of Medicine, Indianapolis, IN, United States

**Keywords:** *Toxoplasma gondii*, DNA repair, DNA damage, drug, therapy

## Abstract

*Toxoplasma gondii* is the causative agent of toxoplasmosis in animals and humans. This infection is transmitted to humans through oocysts released in the feces of the felines into the environment or by ingestion of undercooked meat. This implies that toxoplasmosis is a zoonotic disease and *T. gondii* is a foodborne pathogen. In addition, chronic toxoplasmosis in goats and sheep is the cause of recurrent abortions with economic losses in the sector. It is also a health problem in pets such as cats and dogs. Although there are therapies against this infection in its acute stage, they are not able to permanently eliminate the parasite and sometimes they are not well tolerated. To develop better, safer drugs, we need to elucidate key aspects of the biology of *T. gondii*. In this review, we will discuss the importance of the homologous recombination repair (HRR) pathway in the parasite's lytic cycle and how components of these processes can be potential molecular targets for new drug development programs. In that sense, the effect of different DNA damage agents or HHR inhibitors on the growth and replication of *T. gondii* will be described. Multitarget drugs that were either associated with other targets or were part of general screenings are included in the list, providing a thorough revision of the drugs that can be tested in other scenarios.

## Introduction

Toxoplasmosis is a zoonotic infection caused by the protozoan parasite *Toxoplasma gondii*. This infection is widely distributed in the world, present in 1/3 of the population (Tenter et al., 2000). This wide distribution is based on its life cycle, which has multiple opportunities for transmission to animal hosts. *T. gondii* is capable of infecting all the nucleated cells of mammals and birds, including humans, with cats being the definitive host (Dubey, 2009). Throughout its life cycle *T. gondii* has both sexual (definitive host) and asexual (all hosts) reproduction. The asexual phase consists of two stages: tachyzoites, characterized by a fast-replication rate; and bradyzoites, a tissue encysted stage with a slow division rate that evades both immunity as well as available therapies. In human, the infection naturally occurs orally, either by ingestion of oocysts released by felines present in the soil or water sources (Krueger et al., 2014), or by tissue cysts present in undercooked meats (Wilking et al., 2016; Belluco et al., 2018). Vertical transplacental infection can also occur, called congenital toxoplasmosis which, from a clinical point of view, is of greater importance due to the serious consequences that it may have on the fetus or newborn (Montoya and Remington, 2008). In immunocompetent individuals, the infection is generally mild or asymptomatic during the acute phase. However, it is a major opportunistic infection in the immunocompromised, particularly HIV/AIDS patients. In this case, life-threatening brain lesions can arise if proper treatment is not administered; in addition ocular and pulmonary complications can occur from AIDS-toxoplasmosis (Porter and Sande, 1992; Rabaud et al., 1994). Due to the high incidence of toxoplasmosis in animals of importance for human consumption (Dong et al., 2018; Olsen et al., 2019). *T. gondii* is classified as a food-borne pathogen of high relevance. In fact, the Centers for Disease Control (USA) includes *T. gondii* among the three pathogens, together with *Listeria* and *Salmonella*, as responsible for 70% of foodborne deaths in the United States (Guo et al., 2016).

*Toxoplasma gondii* is considered a parasite of veterinary and medical importance, because it may cause abortion or congenital disease in its intermediate hosts (Sander et al., 2018). Toxoplasmosis has two clinical phases in intermediate hosts: (i) acute, in which the highly replicative tachyzoite stage spreads throughout the body, and (ii) chronic phase, which involves the formation of tissue cysts, preferentially in the nervous system and skeletal muscle, which remain in the host for a lifetime (Delgado Betancourt et al., 2019; Stelzer et al., 2019). Chronic toxoplasmosis has been correlated with a variety of neuropsychiatric disorders that include memory loss, bipolarism, attention deficit hyperactivity disorder and schizophrenia (Chaudhury and Ramana, 2019; Tyebji et al., 2019).

The treatment of the acute phase in cats and dogs is effective and is based on the use of clindamycin, trimethoprim/sulfonamide or azithromycin, either for systemic toxoplasmosis or to abolish oocyst shedding (cats) (Dubey et al., 2009). In humans, the treatment is based on pyrimethamine/sulfadiazine, trimethoprin/sulfamethoxazole or pyrimethamine/clindamycin (Neville et al., 2015). However, it has been observed that about 40% of patients were forced to stop therapy due to low tolerance and serious adverse effects (Porter and Sande, 1992; McLeod et al., 2006; Rajapakse et al., 2013). In addition, drug resistance cases are being reported in some cases of toxoplasmosis (Montazeri et al., 2018). Also, there are no approved therapies for the eradication of the encysted bradyzoite form. Therefore, intense research is focusing on the development of new drugs against both the acute and chronic phases of *T. gondii* infection. In this review, we analyze drugs known to induce DNA damage or block the double strand break (DSB) repair pathway for their therapeutic potential against *T. gondii*. Importantly, many of these drugs have already been approved by the Food and Drug Administration (FDA) or are being examined in clinical trials for other indications (Table S1). We include a list of potential toxic effects of these agents in Table S1 to help prioritize the most promising for future research.

## DNA Repair Machinery

**Figure 1:**
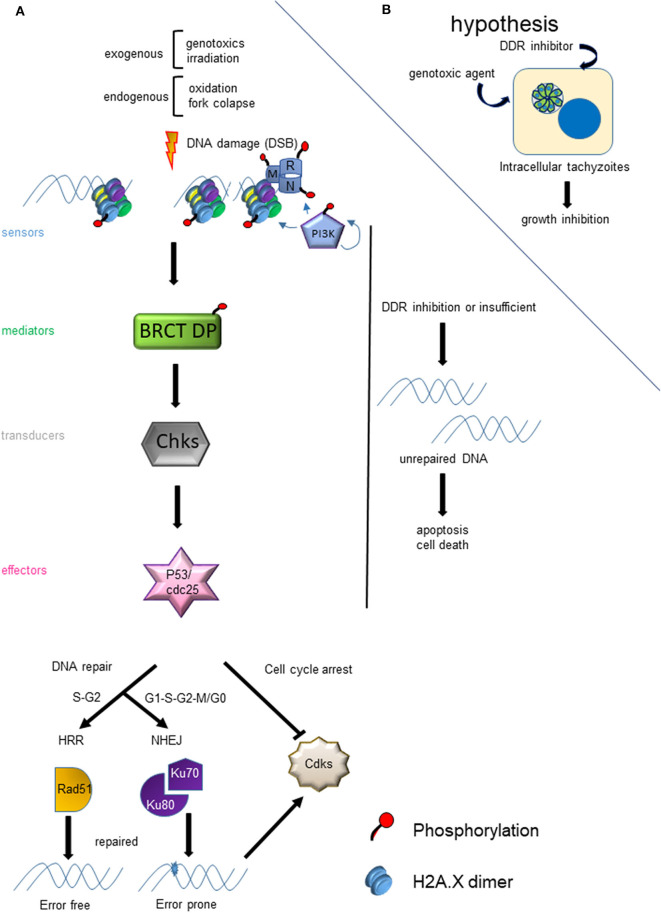
Model of DNA damage response (DDR) due to DSB. **(A)** The panel shows the cascade of events that are triggered by a DSB and the factors that affect the choice of HRR or NHEJ. **(B)** The hypothesis of the use of the HRR pathway as a drug target for the development of new anti-*T. gondii* therapies is graphed.

The genomes of all living cells must be protected from DNA damage, which can be sustained through a wide variety of cellular stress and insults. Lesions in DNA can affect transcription, replication and genomic integrity. DSBs are the most harmful for the cell, which is equipped with different ways to repair the DSB (Sancar et al., 2004). Both exogenous events, like irradiation or genotoxic agents, and endogenous, such as oxidation and fork replication collapse, can result in DSB (Figure 1). Once the DSB is generated, different proteins start to sense the damage and trigger a cascade of events and signals that culminate in the regulation of the cell cycle and transcription until the damage is repaired and DNA replication is allowed to continue (Figure 1). In case the damage cannot be repaired, the cell can enter into apoptosis. Failure to repair DNA properly risks genomic instability, which can lead to degenerative changes.

After sensing the presence of DSB, phosphatidylinositol 3′-kinase–like kinase (PIKK), such as Ataxia telangiectasia mutated (ATM) and DNA dependent protein kinase (DNA-PK), activate (phosphorylate) a series of proteins related to the DNA damage response (DDR). Meanwhile, ATMRad-3-related (ATR), another PIKK, is recruited to stalled replication forks with single strand DNA. When DSB is generated, ATM is the key kinase that phosphorylates sensors such as histone H2A.X, and Mre11, Rad50 and Nbs1 (MRN) complex proteins (Bakkenist and Kastan, 2003) (Figure 1). The phosphorylation of H2A.X in the serine of the SQE motif, referred to as γH2A.X, is an early event and marker of DSB (Rogakou et al., 1998; Sedelnikova et al., 2003). Interestingly, *T. gondii* tachyzoites contain basal levels of γH2A.X even in normal conditions of growth (Dalmasso et al., 2009; Nardelli et al., 2013; Bogado et al., 2014). It is known that intracellular tachyzoites replicate with a doubling time of 5-9 h (Radke et al., 2001), which could be associated with replication stress and fork collapse. This parallels what is seen due to fork collapse under replication-associated DNA stress in cancer cells (Bartkova et al., 2005). The treatment of intracellular tachyzoites with the specific ATM kinase inhibitor is able to block the growth of *T. gondii* and arrest them in G1 (Munera López et al., 2019).

In Eukaryotes, there are two well characterized pathways of DSB repair: Homologous Recombination Repair (HRR) and Non-Homologous End Joining (NHEJ). HRR is an error free pathway that is preferentially activated by DSB occurring during late S and G2 phases (Sancar et al., 2004; Blackwood et al., 2013) (Figure 1). Conversely, NHEJ is an error prone mechanism active across the cell cycle. *T. gondii* has both functional mechanisms (Fenoy et al., 2016). The genome of *T. gondii* encodes near 50% of HRR proteins described in yeast and mammals, suggesting some divergence exists between these pathways, possibly involving parasite-specific components (Fenoy et al., 2016). Additionally, in yeast there is another process of DNA repair based on recombination, called break-induced replication (BIR), which occurs during phase G2 and it is dependent of a single-end DSB (Ait Saada et al., 2018). Another non-canonical process described in yeast and mammals is alternative NHEJ (aNHEJ) or microhomology-mediated end joining (MMEJ). aNHEJ is less faithful than NHEJ and may be associated with deficiencies in the NHEJ pathway (Deriano and Roth, 2013).

Regarding NHEJ, this system presents key proteins such as DNA-PK (geneID 266010), Ku70 (geneID 248160), Ku80 (geneID 312510) although none ortholog of X-ray repair cross-complementing protein 4 (XRCC4), a protein that bridges DNA to DNA Ligase IV, was detected in *T. gondii* (www.toxodb.org) (Munera López et al., 2019). Ku70 appears to be essential for *T. gondii* tachyzoites (Fox et al., 2009), however Ku80 is not and its deletion yielded knockout parasite lines (Δku80) with impaired non-homologous recombination mediated by NHEJ pathway (Fox et al., 2009; Huynh and Carruthers, 2009). *T. gondii* RHΔKU80 showed similar replication rate and virulence as the parental strain, but a marked sensitivity to the genotoxic agents phleomycin and γ-irradiation (Fox et al., 2009). Sidik et al. (2016) have performed a genome wide-screen in which every predicted *T. gondii* gene was assigned fitness score from −6.89 to +2.96, where negative values indicate a disadvantage for the growth of the parasite (www.toxodb.org). In this analysis, DNA-PK has a −3.21 score, inferring that this PI3K is essential and could be a promising target of inhibitors. DNA-PK is recruited to DSB sites by Ku proteins and is able to phosphorylate NHEJ-associated proteins such as Ku, XRCC4 as well as Artemis, ligase IV, and XLF (Deriano and Roth, 2013). In addition, DNA-PK is also involved in HRR, together with ATM kinase (Stiff et al., 2004; Wang et al., 2005), and in the cellular response to hypoxia, different metabolic regulation, innate immunity and transcriptional regulation, among others (Goodwin and Knudsen, 2014). Therefore, the importance of DNA-PK in *T. gondii* viability comparison with Ku80 protein could be due to its pleiotropic role in the parasite biology.

**Figure 2:**
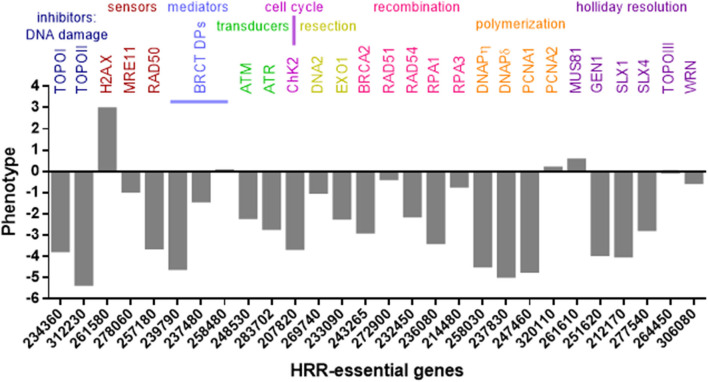
HRR and topoisomerase genes of *T. gondii*. Genes were identified in www.toxodb.org and grouped according to their role in HRR pathway. In addition, phenotype scores associated with fitness were added. The score values were obtained at www.toxodb.org. The scores ranged from −6.89 to +2.96, where negative values indicate that the loss of the gene is disadvantaged or essential for the growth of the parasite.

The *T. gondii* genome harbors 39 coding sequences out of 81 genes described in mammals and yeast responsible for HRR (Fenoy et al., 2016). In general, this pathway is conserved in *T. gondii* (Figure 2), although some important proteins such as Nbs1 and CtIP are absent. Nbs1 and CtIP have a key role at the beginning of HRR because they are part and regulate the activity of Mre11-RAD50-Nbs1 (MRN) complex, a DSB sensor (Figures 1, 2), and end-chain processing (Fenoy et al., 2016). On the other hand, in mammals, BRCT-domain containing proteins BRCA1 and 53BP are involved in regulating the choice of repair pathway: HRR or NHEJ, respectively. Despite their essential roles no homologs for these proteins were detected in *T. gondii*. However, other BRCT domain containing proteins were identified *in T. gondii* without known functions (Fenoy et al., 2016). In *T. gondii*, the HRR pathway is highly efficient as it was observed in *T. gondii* Δku80 lines (Fox et al., 2009; Huynh and Carruthers, 2009). It is worth mentioning that the majority of HRR components of *T. gondii* seem to have essential function, since most show a fitness phenotype score under 0, and even some below−2 (Figure 2). This would indicate that interference of this pathway could significantly alter the proliferation of *T. gondii*, making HRR an important source of novel therapeutic targets.

## DNA Damaging Agents

Nowadays there is a broad spectrum of DNA damage inducers reported in the literature many of which exert their action directly on synthesis and topology of DNA, even some have already been evaluated as anti-toxoplasmic drug targets (Table 1). There is another group of drugs that act indirectly generating oxidative burst that can damage DNA and were assayed against *T. gondii* (Table 2). On the other hand, the methyl methane sulfonate compound (MMS), which methylates DNA at N7-deoxyguanosine and N3-deoxyadenosine, also generates DNA damage in *T. gondii* (Munera López et al., 2019). The damage caused by MMS generally is repaired by the base excision repair (BER) pathway, but it can also lead to fork stalling associated DNA damage (Lundin et al., 2005). In general, MMS is used as an experimental mutagenic agent since it is considered carcinogenic group 2A by the World Health Organization, International Agency for Research on Cancer. In the following sections we will focus on the types of drugs that are related to synthesis and topology of DNA (Table 1).

**Table 1:** DNA damaging agents tested in *T. gondii* models.

**Drug**	**Mechanism**	**Effectiveness**	**References**
Camptothecin	Top1 venom	Moderated	Adeyemi et al., 2019; Munera López et al., 2019
10- Hydroxycamptothecin	Top1 venom	Yes	Adeyemi et al., 2019
Betulin	Top1 inhibitor	No	Adeyemi et al., 2019
Etoposide	Top2 venom	No	Dittmar et al., 2016
Genistin	Top2 inhibitor	No	Adeyemi et al., 2019
Daunorubicin	Top2 inhibitor	Yes	Adeyemi et al., 2019
Trovafloxacin	Top2-a inhibitor	Yes	Khan et al., 1996
Ciprofloxacin	Top2-a inhibitor	No	Khan et al., 1996
Ofloxacin	Top2-a inhibitor	No	Khan et al., 1996
Fleroxacin	Top2-a inhibitor	No	Khan et al., 1996
Temafloxacin	Top2-a inhibitor	No	Khan et al., 1996
Tosufloxacin	Top2-a inhibitor	No	Khan et al., 1996
Enrofloxacin	Top2-a inhibitor	Yes	Barbosa et al., 2012
Gatifloxacin	Top2 inhibitor	Yes	Khan et al., 2001
Doxorubicin	Top2 venom/DNA adducts	No	Dittmar et al., 2016
Aclarubicin	Top inhibitor/ oxidative DNA damage	No	Adeyemi et al., 2019
Artemisinin	Top1 inhibitor	Yes	Jones-Brando et al., 2006
Artemether	Top1 inhibitor	Yes	Jones-Brando et al., 2006
Artemisinin derivatives	Top1 inhibitor	Yes	D'Angelo et al., 2009; Schultz et al., 2014
Harmane	Top1 inhibitor	Yes	Alomar et al., 2013
Harmine	Top1 inhibitor	Yes	Alomar et al., 2013
nor-Harmane	Top1 inhibitor	Yes	Alomar et al., 2013
Fluorouracil (5-FU)	thymidylate synthase inhibitor	Yes	Harris et al., 1988
Vincristine	DNA intercalation	No	Dittmar et al., 2016
Fluphenazine	DNA intercalation	Yes	Goodwin et al., 2011; Murata et al., 2017
Thioridazine	DNA intercalation	Yes	Goodwin et al., 2011
Trifluoperazine	DNA intercalation	Yes	Goodwin et al., 2011
Hycantone	DNA intercalation	Yes	Murata et al., 2017
Phleomycin	DNA intercalation	ND	Messina et al., 1995
Mitomycin C	DNA intercalation	Yes	Adeyemi et al., 2019
Thiosemicarbazones	ribonucleotide reductase inhibitor	Yes	Tenório et al., 2005
Hydroxyurea	ribonucleotide reductase inhibitor	Yes	De Melo et al., 2000
Cytarabine	DNA polymerase inhibitor	no	Adeyemi et al., 2019
Methyl Methanesulphonate (MMS)	alkylates DNA bases	Yes	Munera López et al., 2019

**Table 2:** Non direct DNA damaging agents used in *T. gondii* models.

**Drug**	**Mechanism**	**Effectiveness**	**References**
Resveratrol	oxidative DNA damage/ DNA binding	Yes	Adeyemi et al., 2019; Chen et al., 2019
Tamoxifen	oxidative DNA damage	Yes	Dittmar et al., 2016
Butein	oxidative DNA damage	No	Murata et al., 2017; Adeyemi et al., 2019
Amphotericin	oxidative DNA damage	No	Adeyemi et al., 2019
Menadione	oxidative DNA damage	No	Adeyemi et al., 2019
Capsiacin	oxidative DNA damage	No	Adeyemi et al., 2019
Sertraline	neurotransmission/ oxidative damage	Yes	Dittmar et al., 2016
Andrographolide	undetermined	No	Adeyemi et al., 2019
Chloroquine	undetermined	Yes	Adeyemi et al., 2019
Loperamide	gut opiate receptor	Yes	Dittmar et al., 2016
PurvalaNol A	CDks	Yes	Dittmar et al., 2016
SB 218078	ChK1 inhibitor	No	Dittmar et al., 2016
Valproic acid	oxidative DNA damage	Yes	Jones-Brando et al., 2003

### Topoisomerase Inhibitors

Interference with DNA replication perturbs progression of the cell cycle and would result in poor proliferation. Topoisomerases are responsible for removing the negative supercoiling that occurs during advances of replication forks or transcription, but also are involved in recombination process (Wang, 2002). Topoisomerases I (Top1) and II (Top2) are enzymes that resolve torsional stress and supercoiled structures on replicating DNA. In *T. gondii*, both Top1 and Top2 have shown a score associated to a high degree of essentiality (Figure 2). Top1 mediated-DNA relaxation involves the cut of one DNA strands, its rotation and religation. Top2 enzymes also bind to DNA (G-segment), generating a DSB through which another segment (T-segment) of the dsDNA passes, crossing the DSB, in such a way that the ends of G-segment are then re-ligated (Jain et al., 2017). Several Top1 and Top2 inhibitors have emerged in recent years for use in anti-cancer chemotherapies (Jain et al., 2017). This type of drugs can be classified as inhibitors, when they block the enzyme activity or binding to DNA; or venoms, those that stabilize the topoisomerase-DNA complex.

There are several Top1 and Top2 inhibitors or venoms that show anti-*T. gondii* effect (Table 1). A plant alkaloid, camptothecin (CPT), was one of the first Top1 inhibitors, but other camptothecin derivatives were also studied: topotecan and irinotecan, both approved by the US Food and Drug Administration (FDA) in 1996 (Thomas and Pommier, 2019). Since CPT is insoluble, CPT derivatives topotecan (9-dimethyl-aminomethyl-10-hydroxycamptothecin), irinotecan (7-ethyl-10-Hydroxycamptothecin) and others, contain solubilizing groups added to the core CPT (Musiol, 2017). Moreover, another camptothecin analog, 10-Hydroxycamptothecin, also shows a stronger anti-tumor activity and lesser toxicity than camptothecin (Hu et al., 2011). Recently, CPT has been shown to have moderate anti-*T. gondii* activity in two independent publications. In one of them, CPT showed a IC_50_ value near to 5 μM, a dose that leads to a 40% viability of the host cell (Munera López et al., 2019). In the other, Adeyemi et al. (2019) performed a screening of 62 compounds at a concentration of 1.5 μg/mL where CPT (4.3 μM) showed an inhibition of 37% in proliferation and 27% viability of the host cell. Based on these findings, CPT would not present a great value as an anti-*T. gondii* drug even having been approved by the FDA. However, it could be useful as a model to generate DSB during parasite replication. In fact, increased levels of phosphorylation to *T. gondii* H2A.X (γH2A.X) were observed in intracellular tachyzoites treated with CPT (Munera López et al., 2019). In the screening performed by Adeyemi et al. (2019), they detected that the analog of camptothecin, 10 hydroxycamptothecin, showed no host cell toxicity and inhibited *T. gondii* proliferation by 58.16% at a dose of 1.5 μg/mL (4.1 μM). This result suggests that this and other camptothecin analogs could be analyzed in greater depth in their potential as candidates for anti-*T. gondii* therapy.

Another group of eukaryotic topoisomerase inhibitors are quinolones and fluorquinolones that were first identified as anti-bacterial topoisomerase IV and DNA gyrase drugs (Gootz et al., 1990; Brighty and Gootz, 1997; Jorgensen et al., 2000). Among them, enrofloxacin, ciprofloxacin, fleroxacin, temafloxacin, tosufloxacin, ofloxacin, trovafloxacin and gatifloxacin have been tested in *T. gondii* models (Khan et al., 1996; Barbosa et al., 2012) and reviewed by Alday and Doggett (2017). Only enrofloxacin, trovafloxacin and gatifloxacin showed anti-*T. gondii* activity (Table 1). Trovafloxacin was effective both *in vitro* and *in vivo*. In the latter, a dose of 100 mg/kg per day during 10 days postinfection with virulent strain RH showed significant protection of infected mice against death (Khan et al., 1996). In addition, lower doses of trovafloxacin combined with other drugs such as clarithromycin, pyrimethamine, or sulfadiazine had a synergistic effect against *T. gondii* infection (Khan et al., 1997). The same authors tested a new fluoroquinolone, gatifloxacin, which also had partial protection *in vivo* when administered alone and a synergistic effect in combination with pyrimethamine (Khan et al., 2001). The mechanism of action of these fluoroquinolones against *T. gondii* was not elucidated. Ciprofloxacin did not show any anti-*T. gondii* effect *in vitro* at concentrations from 0.625 to 10 μg/mL (Khan et al., 1996). However, treatment with 25 μM (near 8 μg/mL) ciprofloxacin reduced the amount of apicoplast DNA in *T. gondii*, suggesting that an apicoplast (prokaryotic-like) DNA gyrase could be a target (Fichera and Roos, 1997). However, it cannot be ruled out that these fluoroquinolones also target eukaryotic topoisomerases. Ciprofloxacin and trovafloxacin have been shown as weak inhibitors of topoisomerase II-α (Gootz et al., 1990; Poulsen et al., 2014).

Artemisinin, a sesquiterpene lactone, and its derivative artemether have shown activity toward tumors and target topoisomerase I whereas artesunate, a semisynthetic derivative of artemisinin, inhibits topoisomerase II (Kadioglu et al., 2017). In an *in vitro* study, artemisinin and artemisinin derivatives (2a, 2b, 2c, 2d, deoxy-2a and artemether) displayed anti-*T. gondii* activity, with artemether, 2c and 2b being the most effective drugs on the basis of their therapeutic indices (Jones-Brando et al., 2006). Since artemisinin and some derivatives have shown neurotoxicity, other less toxic compounds (C-10 unsaturated, carba-linked) were synthesized and tested *in vitro* (D'Angelo et al., 2009). The majority inhibited host cell invasion and/or tachyzoite replication and growth, but none of them were more effective than artemether (D'Angelo et al., 2009). Later, Schultz et al. (2014) tested different artemisinin derivatives, including artemether and the novel LEW3-27 and CPH4-136 *in vivo*. They observed that only the C-10 carba-linked, unsaturated artemisinin derivative CPH4-136 produced a moderate effect on mouse survival of acute infection, but it displayed a 40% reduction of brain cyst burden in an experimental model of murine chronic infection. In *P. falciparum*, artemisinin appears to affect many systems, including antioxidant defense, hemoglobin degradation, glycolysis, chaperone machinery, and enzymes of purine and pyrimidine synthesis, among others, but not with topoisomerase (Ismail et al., 2016). Recently, a protease (DegP2) belonging to the high temperature requirement A family (HtrA), predicted as a mitochondrial protein in toxodb, and ARK kinase, predicted as a nucleolar kinase in toxodb, arose as artemisinin targets (Rosenberg et al., 2019). Together, these studies suggest that the aforementioned drugs have complex mechanisms of actions that may involve multiple targets.

Another group of compounds that has been shown to intercalate with DNA and inhibit topoisomerase are β-carbolines and derivatives (Cao et al., 2007). However, β-carbolines could also be multitargeting drugs, inhibiting some kinases such as cyclin dependent kinases and PI3K (Song et al., 2002; Zhang et al., 2016). Moreover, it has recently been observed that β-carboline harmine does not induce DNA damage (Geng et al., 2018). Treatment of intracellular tachyzoites with harmane, norharmane, and harmine reduced parasite replication and growth at concentrations below 12.5 μM (Alomar et al., 2013). However, this study pointed out that the three β-carbolines also affected *T. gondii* host cell invasion, suggesting that other targets unrelated to DNA topology could be involved. In fact, harmine was identified as a potent anti-plasmodial drug targeting parasite Hsp90 (Shahinas et al., 2012). *Plasmodium falciparum* and *T. gondii* Hsp90 are highly identical at primary sequence level, and *T. gondii* Hsp90 has also shown to be involved in parasite cell cycle and differentiation (Echeverria et al., 2005).

Finally, other topoisomerase inhibitors were assayed against toxoplasmosis in different experiments (Dittmar et al., 2016; Adeyemi et al., 2019): betulinic acid, etoposide, genistin, daunorubicin, doxorubicin and aclarubicin. Evidence of their inhibitory effects against topoisomerases have been observed by different authors (Bodley et al., 1989; Chowdhury et al., 2002; Larsen et al., 2003; Mizushina et al., 2013; Lee et al., 2016; Amaral et al., 2017). It is important to note that only daunorubicin showed effects against *T. gondii* with an inhibition value of 41.6% and host cell viability of 62.27% at a concentration of 1.5 μg/mL (Adeyemi et al., 2019). Daunorubicin is an anthracycline compound, such as doxorubicin and aclarubicin, that has shown to generate DNA breaks and DNA-protein crosslinking at lower concentrations, suggesting topoisomerase II inhibition (Ciesielska et al., 2005). Daunorubicin is more lipophilic than doxorubicin (Gallois et al., 1998), a property that could be advantageous in the trespassing of the multiple membrane barriers of infected host cells. However, it should be used with caution because daunorubicin, as well as other anthracyclines, has shown cardiotoxicity (Aubel-Sadron and Londos-Gagliardi, 1984).

### DNA Intercalating Compounds

Other DNA damaging agents intersperse within the DNA molecule. A single intercalation is usually not enough to cause damage, however major structural changes might lead to inhibition of replication and transcription processes. Most intercalating agents do not produce DSB in purified DNA, suggesting that, *in vivo*, the stabilization of DNA-intercalating complex disturbs the activity of topoisomerase enzyme (Tomczyk and Walczak, 2018). For this reason, many DNA intercalating compounds are also associated with topoisomerase inhibition. Table 1 summarizes several damage intercalating agents that were tested against *T. gondii*.

Fluphenazine, trifluoperazine, and thioridazine are phenothiazine drugs widely used as antipsychotics in schizophrenia (Matar et al., 2014). Phenothiazines have been shown to associate externally to plasmid DNA, nicking the supercoiled plasmid under photoinduction (Viola et al., 2003). Because chronic toxoplasmosis has been linked to different mental health disorders (Tyebji et al., 2019), among them schizophrenia, many authors tested the effect of antipsychotic drugs against *T. gondii* (Holfels et al., 1994; Jones-Brando et al., 2003; Goodwin et al., 2011). Fluphenazine has shown good activity against *T. gondii* growth (Jones-Brando et al., 2003; Fond et al., 2014; Murata et al., 2017). Fluphenazine, trifluoperazine, and thioridazine had IC_50_ values *in vitro* of 1.7, 3.8, and 1.2 μM, respectively (Goodwin et al., 2011). Neither fluphenazine nor thioridazine blocked cyst formation in an experimental model in mouse (Saraei et al., 2016).

Phleomycin is an antibiotic of bacterial origin whose mechanism of action is through intercalating into DNA (Wheatley et al., 1974). This antibiotic affects *T. gondii* replication and has been used as a selection marker of transfected tachyzoites but not for therapeutic purposes. The treatment of extracellular tachyzoites with high doses of phleomycin reduces their ability to proliferate when these treated parasites are used to infect host cell monolayers (Messina et al., 1995; Soldati et al., 1995). The resistance cassette used in *T. gondii* vectors encodes BLE protein from Tn5 transposon, which binds to phleomycin in a 1:1 complex, neutralizing its toxicity.

Mitomycin C and hycanthone showed an anti-*T. gondii* effect but vincristine was not effective enough to be selected for further studies (Dittmar et al., 2016; Murata et al., 2017; Adeyemi et al., 2019). Mitomycin C is a well-known DNA binding compound that induces DNA-DNA interstrand crosslinking (Poll and Arwert, 1985). Hycanthone is a potent mutagen that was demonstrated to intercalate DNA and generate single stranded DNA (Bases et al., 1978). Vincristine was shown to preferentially bind to double stranded DNA, although it showed higher affinity for chromatin than to naked DNA, suggesting an intercalation between the phosphate sugar backbone and histones (Mohammadgholi et al., 2013). Mitomycin C is a drug used to treat some cancers such as breast, bladder, gullet (esophagus), stomach, pancreas, lung, anal and liver cancers. In this regard, anti-*T. gondii* ability of Mitomycin C was evaluated showing 45.06% of tachyzoite growth inhibition and 103% of host cell viability at 1.5 μM/mL (Adeyemi et al., 2019). The complementation of an *Escherichia coli* mutant lacking recombinase enzyme RuvC with the ORF expressing the *T. gondii* TgDRE enzyme recovered bacterial resistance to mitomycin C and UV-light (Dendouga et al., 2002). The TgDRE gene is annotated as a G-patch domain containing protein in toxodb (TGME49_214820) and its contribution to *T. gondii* lytic cycle fitness has a phenotype score of −4.12, suggesting that it is likely to be essential. It will be interesting to study in the future if TgDRE also confers mitomycin C resistance (or to other DNA damaging agents) in *T. gondii*.

### DNA Synthesis Inhibitors

The synthesis of DNA requires many enzymes, including DNA polymerase and those that synthesize deoxyribonucleotides (e.g., ribonucleotide reductase). The inhibition of some of these interferes with the process by stalling the replication fork, which can consequently generate single strand DNA or even DSB (Abraham, 2001).

Among ribonucleotide reductase inhibitors, hydroxyurea (HU) possesses anti-*T. gondii* activity affecting its proliferation at 4 mM and induces morphological changes in intracellular tachyzoites (De Melo et al., 2000). In accordance with previous observations, the same dose leads to an arrest in G1 cell division after 24 h of treatment in synchronized intracellular tachyzoites (Munera López et al., 2019). Moreover, the growth of *T. gondii* is reduced by 50% in the presence of 150 μM HU (Munera López et al., 2019). However, at that dose, HU showed no synergism with KU-55933, an inhibitor of ATM kinase, an enzyme key to the development of HRR.

Another class of ribonucleotide reductase inhibitors with antitumor activity are thiosemicarbazones. The mechanism of action is suggested to be based on their metal chelating nature. Since ribonucleotide reductase enzyme contains a diferric center, thiosemicarbazones change the rate-limiting step in DNA synthesis (Kalinowski et al., 2009). Tenório et al. (2005) synthesized a wide variety of thiosemicarbazone derivatives from two series: (i) thiosemicarbazone compounds 2 and (ii) 4-thiazolidinone derivatives 3. These compounds significantly reduced the number of infected host cells in doses of 1 mM, even more than 4 mM HU. Based on this study, new 4-thiazolidinone derivatives were synthesized which also were very effective against *T. gondii* but less toxic to the host cell (de Aquino et al., 2008; Liesen et al., 2010).

Fluorouracil (5-FU) generates damage in two ways, one by inhibiting thymidylate synthase, a rate-limiting enzyme for DNA synthesis, and the other through its metabolic conversion to FdUTP which is misincorporated into the DNA molecule (Longley et al., 2003). It has long been known that *T. gondii* is susceptible to 5-FU. In 1977, Pfefferkorn and Pfefferkorn (1977) had characterized strains of *T. gondii* resistant to different anti-toxoplasmic agents, including 5-FU. Several years later, the effect of 5-FU on tachyzoite replication in a myoblast cell model was further tested by incorporating [H3]uracil. 5-FU produced a significant replication inhibition from 0.01 μg/mL, being very evident at 0.1 μg/mL, which is below the toxicity concentration for the host cell (Harris et al., 1988). Interestingly, this drug showed a synergistic effect with pyrimethamine. Use of 5-FU was not effective against the cyst *in vitro* studies (Huskinson-Mark et al., 1991).

Cytarabine is a pyrimidine nucleoside-based anticancer drug that, after three phosphorylation steps, inhibits DNA polymerase by competing with the natural substrate, resulting in DNA synthesis inhibition (Chhikara and Parang, 2010). This drug was tested against toxoplasmosis *in vitro* and it only reduced intracellular tachyzoite growth by 14% (Adeyemi et al., 2019). It is worth mentioning that in future studies the anti-toxoplasmic capacity of cytarabine could be tested in combination with other anticancer agents, such as daunorubicin, doxorubicin, thioguanine, or vincristine.

## HRR Inhibitors

The Homologous Recombination Repair (HRR) pathway is well conserved in *T. gondii*, with the majority of related genes exhibiting a negative fitness value, thus highlighting their functional relevance during the tachyzoite replication (Figure 2). HRR is a highly complex process, requiring specific proteins at each step (Fenoy et al., 2016) that may be druggable (Carvalho and Kanaar, 2014; Jekimovs et al., 2014; Velic et al., 2015). HRR starts with sensor proteins that detect the DSB and promote the resection of DNA where different exonucleases participate to generate a long single strand (Figures 1, 2). For example, the exonuclease activity of Mre11 is inhibited by mirin and its amino substituted derivate, PFM39, while N-alkylated derivatives PFM01 and PFM03 block its endonuclease function (Velic et al., 2015). At the same time during DSB sensing, checkpoints are activated and the cell cycle is stopped. ATM, ATR, and DNA-PK belong to PIKK family, and, together with checkpoint kinases (Chk1 and Chk2), are susceptible to inhibition, blocking DNA damage repair (DDR) (Shibata et al., 2014). Once the single strand is generated after DNA resection, the filament recruits the RAD51 recombinase and other proteins that seek a template in which DNA repair associated DNA polymerases synthesize new strands; this process results in Holliday-junction binding, which is resolved by different helicases.

Rad51 seems to be a great pharmacological target due to its central role in the parasites HRR pathway, but structural and enzymatic studies are needed because is highly ubiquitous in the human host, so specific inhibitors have to be achieved to target the parasite protein instead of the human RAD51 (HuRad51) (Kelso et al., 2017). Recent studies are investigating the HuRAD51 inhibitors in parasites. The molecule 4,4′- diisothiocyanostilbene-2,2′-disulfonic acid (DIDS) in humans inhibits the HuRad51 binding to dsDNA and ssDNA (Ishida et al., 2009). DIDS also has effect in *Entamoeba histolytica* RAD51 inhibiting its DNA binding, and in *Entamoeba invadens* attenuates the encystations process (Kelso et al., 2017). Plasmodial Rad51 is inhibited by compound B02 (Vydyam et al., 2019). While β-carbolines were suggested to be possible DNA intercalating agents or topoisomerase inhibitors (see above), it has also been observed that they block HRR, affecting the recruitment of RAD51 by a route not yet described (Zhang et al., 2015). More studies should be done to check if β-carbolines affect the HRR pathway in *T. gondii*.

### PIKK Kinase Inhibitors

Given their central role in the cascade of DNA damage repair events, the PIKKs are good candidates as drug targets (Table 3). To note, there are several kinases (e.g., p38 MAPK) whose inhibition can block HRR indirectly, but here we only focus on PIKK inhibitors. *T. gondii* appears to have the three PIKKs associated with DSB repair: ATM, ATR, and DNA-PK (Fenoy et al., 2016; Munera López et al., 2019). Caffeine is a broad-spectrum kinase inhibitor compound capable of inhibiting the afore mentioned PIKKs (Sarkaria, 2003; Bode and Dong, 2007). When caffeine is applied to infected HFF cultures, it reduces cell growth with an IC_50_ value 370 μM with a LD_100_ of 800 μM (Munera López et al., 2019). Similarly, the isoflavonoid quercetin and its relative LY294002 compound were shown to competitively inhibit the binding of ATP to PI3K and DNA-PK (Izzard et al., 1999). Both were assayed by Dittmar et al. (2016), but none of them were selected for further analysis. The compound NU7026 (DNA-PK inhibitor) also showed little value as an anti-*T. gondii* drug in a first screening (Dittmar et al., 2016). NU7026 is a specific DNA-PK inhibitor based on small molecule NU7741, which displays anti-tumoral activity (Tsai et al., 2017). Moreover, NU7026 at 10 μM shows a synergistic effect with γ irradiation in neuroblastoma cells (Dolman et al., 2015), at a higher dose than that used by Dittmar et al. (2016). For this reason, it would be prudent to reexamine if these DNA-PK inhibitors could affect *T. gondii* replication either alone or in combination with DNA damaging agents.

**Table 3:** HRR inhibitors used in *T. gondii* models.

**Drug**	**Mechanism**	**Effectiveness**	**References**
KU-55933	ATM inhibitor	Yes	Munera López et al., 2019
NU 7026	DNA-PK inhibitor	No	Dittmar et al., 2016
Quercetin	PI3K/DNA-PK inhibitor	No	Dittmar et al., 2016
LY 294002	PI3K/DNA-PK inhibitor	No	Dittmar et al., 2016
Caffeine	Kinase inhibitor	Yes	Munera López et al., 2019
SAHA/vorionstat	HDACi	Yes	Strobl et al., 2007
Scriptaid	HDACi	Yes	Strobl et al., 2007
Trichostatin A	HDACi	Yes	Strobl et al., 2007
Sodium butyrate	HDACi	No	Strobl et al., 2007
Valproic acid	HDACi	No	Strobl et al., 2007
4-phenylbutyrate	HDACi	No	Strobl et al., 2007
Curcumin	HDACi	No	Adeyemi et al., 2019
Nicotinamide	SIRTi	No	Strobl et al., 2007
Resveratrol	SIRT1a	Yes	Adeyemi et al., 2019; Chen et al., 2019
Harmine	ND	Yes	Alomar et al., 2013

**Figure 3:**
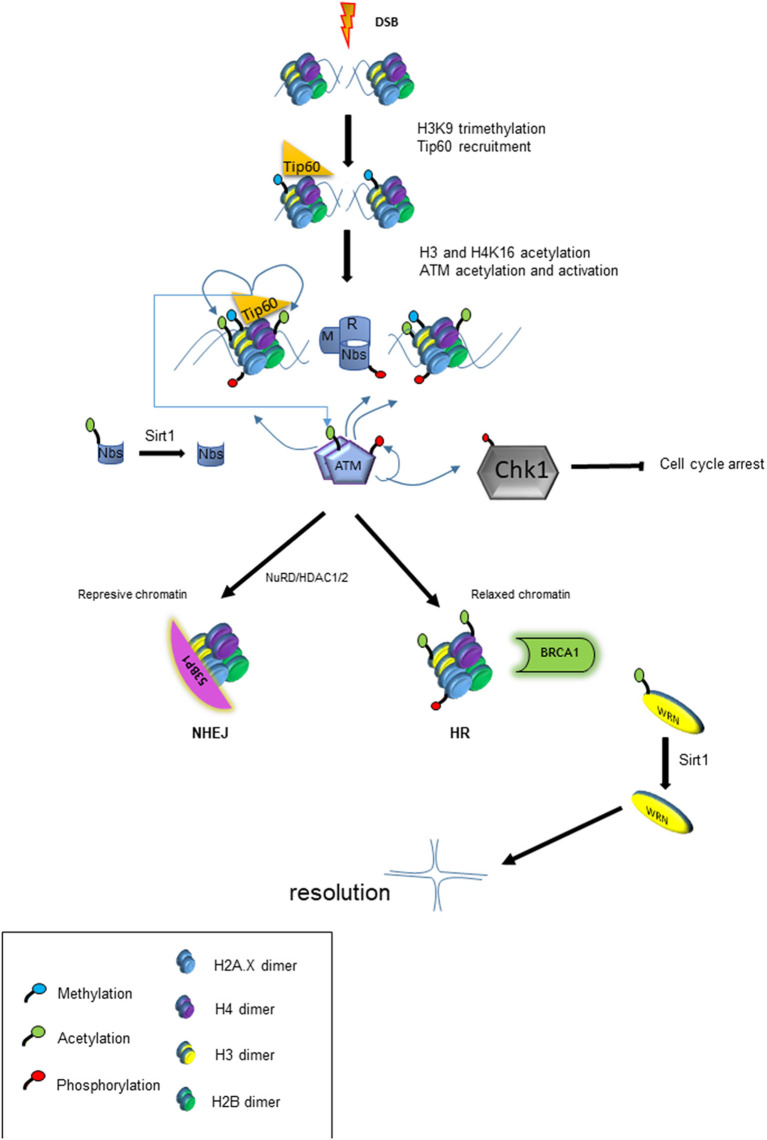
Model of chromatin modification and its impact on DDR. The panel shows the cascade of events that lead to chromatin modification and other post-translational modifications (PTM) that trigger HRR or NHEJ response.

KU-55933 is a ATM kinase inhibitor that has been shown to effectively block the intracellular growth of tachyzoites (Munera López et al., 2019). ATM kinase has a central role in activating the response to the DSB, acting very early in signal transduction to begin repair of damaged DNA. ATM-phosphorylated proteins in mammals include histone H2A.X (to give γH2A.X), p53, Mdm2, Chk1, Chk2, Nbs1, Brca1 among others (Boohaker and Xu, 2014; Guleria and Chandna, 2016). In mammals, the presence of DSB causes changes in chromatin in which H3K9 is methylated (H3K9me3), recruiting Tip60, a Histone acetyl transferase belonging to Myst family, which acetylates histones and ATM (Figure 3). Acetylation of ATM is an important postraductional modification (PTM), required for its autophosphorylation, monomerization, and activation (Sun et al., 2005; Guleria and Chandna, 2016). This pathway appears to be present in *T. gondii* since overexpression of TgMYST-B confers greater resistance of tachyzoites to the DNA damaging agent methyl methanosulfonate (MMS) (Vonlaufen et al., 2010). Furthermore, over-expression of functional TgMYST-B reduces the replication rate of intracellular tachyzoites, strongly suggesting that acetylation of ATM and its subsequent activation results in phosphorylation of checkpoint kinases, an effect that could be reversed by adding KU-55933.

Later, Munera López et al. (2019) observed that the application of KU-55933 to infected host cell cultures blocked the replication and growth of intracellular tachyzoites with an IC_50_ of 2.15-2.49 μM, while even higher concentrations as 10 μM did not affect host cell viability. It is worth mentioning that KU-55933 blocks the induction of phosphorylation on H2A.X (γH2AX) due to CPT treatment (Munera López et al., 2019). These data suggest that KU-55933 affects a kinase able to phosphorylate H2A.X upon DNA injury. Human ATM has a molecular weight of 350–370 kDa, which is similar to yeast (Tel1) near 320 kDa; interestingly, *T. gondii* ATM has a predicted mass of only 246 kDa (Munera López et al., 2019). The homology within *T. gondii* ATM is largely limited to the kinase and FATC domains. Notably, searches of the *Plasmodium* database (plasmodb) only retrieves one PI3K (Fenoy et al., 2016). However, that *Plasmodium* kinase does not possess the FATC domain and is a kinase exported to the host erythrocyte with a role in hemoglobin trafficking (Vaid et al., 2010). In conclusion, *T. gondii*, seems to retain the signaling pathway using an ATM-like enzyme, which would have substantial differences with human ATM, making it possible to be targeted by specific drugs.

### HDAC/KDAC Inhibitors

Protein acetylation has gained great interest due to its regulatory role in a wide variety of biological processes. Initially, studies were focused on histones, however this PTM is present on many other proteins, including a number involved in DNA repair like chaperone HSP90 and ATM kinase. The acetylation of proteins is mediated by lysine acetyltransferases (HAT/KAT) while deacetylation is performed by lysine deacetylases (HDAC/KDAC). Among the processes controlled by this PTM are epigenetic regulation of gene expression and protein-protein interactions, which in turn could facilitate DNA repair either through HRR or NHEJ (Koprinarova et al., 2011; Roos and Krumm, 2016). *T. gondii* contains GCN5 and MYST family KATs as well as several HDAC proteins (Vanagas et al., 2012).

HDACs are associated with the regulation of numerous genes that code for proteins involved in DDR. They are also linked to the direct deacetylation of lysines on many of these proteins (Figure 3). As already mentioned above, one of the first steps in the response to DSB damage is the activation of ATM kinase, which depends on acetylation by Tip60. Prior to the DSB resulting from fork collapse, the fork stalling generates ssDNAs which recruit ATR to trigger checkpoints activation. ATR binds to DNA in a protein complex (CHD4) together with HDAC1 and HDAC2 (Schmidt and Schreiber, 1999). Then chromatin is modified during the DDR process being modulation of H4K16ac and H4K56ac marks of key modification. The presence of HDAC1 and 2 at the site of damage leads to the deacetylation of H4K56ac and H4K16ac promoting repair via NHEJ (Miller et al., 2010). The mark H4K16ac prevents BRCT containing protein 53BP from binding to the chromatin and facilitates linkage of another BRCT protein (BRCA1), driving the HRR pathway (Vanagas et al., 2019). On the other hand, the inhibition of HDAC1 and 2 renders higher levels of acetylated Ku70, which does not form Ku70/Ku80 hetero-dimerization, an essential step for NHEJ repair; this therefore sensitizes the cell to genotoxic agents such as bleomycin, doxorubicin, and etoposide (Chen et al., 2007). Interestingly, sirtuins such as Sirt1 can deacetylate the Nbs1 protein, one of the components of MRN sensor complex, which is important for their phosphorylation and concomitant recruitment of DNA repair factors (Yuan et al., 2007) (Figure 3). Later it was shown that one of the roles of Sirt1 is through the acetylation of the RecQ WRN helicase, which is involved in Holliday junction (Uhl et al., 2010). In summary, modulation of proteins through acetylation is a critical part of DSB repair, suggesting that the inhibition of HDAC may result in deficiencies in the DNA damage response. Table 3 lists HDAC inhibitors (HDACi) tested to date for their anti-*T gondii* action.

Suberoylanilide hydroxamic acid (SAHA) is a pan-HDACi that has shown antitumor activity by regulating HRR-associated genes such as BRCA1 (Konstantinopoulos et al., 2014) and also by affecting the deacetylation of Ku70 (Kerr et al., 2012). SAHA inhibited the growth of *T. gondii* tachyzoites with an IC_50_ 83 nM, a concentration 10-200 lower than that required to have an *in vitro* antitumor effect (Strobl et al., 2007). In the same work, other HDACi proved to be very effective in blocking the growth of *T. gondii* (scriptaid, IC_50_ 39 nM; trichosan A, IC_50_ 41 nM). Interestingly, low doses of these HDACi, between 1 and 50 nM, stimulated the number of tachyzoites in *in vitro* cultures (Strobl et al., 2007). Other HDACi, such as the carboxylic acids (valproic, butyric and derivatives), showed a reduced inhibitory potential with IC_50_s between 1 to 5.35μM, while the inhibitor of sirtuin 1, nicotinamide, showed no antitoxoplasmic capacity (Strobl et al., 2007). The plant polyphenol curcumin is another pan-HDAC inhibitor, which also inhibits repair of DSB in yeast (Wang et al., 2015). However, its application in infected cultures of *T. gondii* did not inhibit the replication of tachyzoite in a drug screen (Adeyemi et al., 2019). It is worth noting that the concentration of curcumin tested against *T. gondii* was 2.71 μM, whereas in the yeast model 50 μM was used in combination with MMS. In yeast, curcumin inhibits HRR presumably by promoting the degradation of RAD52 recombinase, which does not seem to be conserved in *T. gondii* (Fenoy et al., 2016).

Resveratrol (RSV) is a natural phenol with a potent antioxidant activity (Murcia and Martínez-Tomé, 2001). However, it has been shown to modulate a great variety of cell-signaling pathways and interact with numerous molecular targets (Harikumar and Aggarwal, 2008). Notably, it was demonstrated that resveratrol is an activator of type III HDAC (Kaeberlein et al., 2005) but, in certain tumor lines, RSV downregulates DNA methyltransferases (DNMT), inhibits HDACs type I or II, and activates HAT, impacting on epigenetic marks in different ways (Chatterjee et al., 2019; Hu et al., 2019; Izquierdo-Torres et al., 2019). RSV inhibits growth of *T. gondii in vitro* with an IC_50_ of 54 μM after 24 h of treatment on extracellular tachyzoites (Chen et al., 2019). To note, extracellular tachyzoites do not replicate, suggesting that antioxidant activity may have been responsible for this effect. Adeyemi et al. (2019) also found that RSV had anti-*T. gondii* activity, with an IC_50_ of 1.03 μg/ml (4.4 μM) against intracellular tachyzoites. They also demonstrated RSV did not affect the viability of HFF cells, even at 2 μg/ml. The mechanism for RSV is likely to be complicated because it appears to have multiple targets. RSV also targets tyrosyl-tRNAsynthetase (TyrRS), involved in protein synthesis, and TyrRS is linked to the regulation of genes associated with HRR (Cao et al., 2017). RSV causes inactivation of TyrRS, its translocation to the nucleus and, therefore, inhibits HRR (Gao et al., 2019).

## Conclusions and Future Perspectives

*T. gondii* uses both NHEJ and HRR pathways, but evolutionary divergences such as of the lack of key proteins, suggest differences that may have therapeutic value. HRR pathway seems to be relevant even under normal growth conditions, suggesting that across the lytic cycle *T. gondii* is exposed to situations that generate DSB, like collapse of replication forks. In the future it would be important to better understand the dynamics of the HRR process under normal versus DNA damage conditions. It is also of interest to identify the proteins critical to the cellular decision between using the NHEJ or HRR pathways.

Like other organisms, *T. gondii* requires high fidelity of DNA replication during proliferation. Drugs that induce DNA damage could be effective against the parasite, but that effectiveness may be limited if DNA repair mechanisms are intact. Combined therapies that induce DNA damage and neutralize repair enzymes may be required. A better approach could be to target topoisomerases and other enzymes (e.g., ribonucleotide reductase) related to the synthesis of the new DNA chain.

The fact that HRR proteins seem to be crucial in *T. gondii* and that some have structural divergences, make them promising candidates as drug targets. The differences between *T. gondii* and host enzymes can provide valuable information to obtain drugs that specifically block *T. gondii* without affecting the human or animal. Among the compounds analyzed here some are multitargets and often exert different actions depending on the organism. It would be best to identify specific and effective drugs against a single target to avoid unexpected adverse effects on the host. In addition, there are many HRR druggable targets that were not assayed against *T. gondii* yet, but could be excellent candidates. Moreover, the HRR pathway in *T. gondii* may have other proteins whose identification and analysis in the future will allow us to gain knowledge in the HRR mechanisms and could be novel drug targets.

It is clear that the DNA repair pathway is a promising source of new therapeutic targets while offering an important opportunity to generate knowledge about this biological process. Finally, this mechanism allows the combined use of DNA damaging agents and DNA repair inhibitors, thereby increasing the efficiency of therapies.

## Author Contributions

SA, LV, and DR designed the review structure, searched the overall data in the bibliography, and wrote the first version of the review. AS, CC, and WS collaborated in writing some specific items and read and corrected the latest versions of the manuscript. All authors contributed to the article and approved the submitted version.

## Conflict of Interest

The authors declare that the research was conducted in the absence of any commercial or financial relationships that could be construed as a potential conflict of interest.
